# Smooth Normative Brain Mapping of Three‐Dimensional Morphometry Imaging Data Using Skew‐Normal Regression

**DOI:** 10.1002/hbm.70185

**Published:** 2025-03-03

**Authors:** Marco Palma, Shahin Tavakoli, Julia Brettschneider, Ana‐Maria Staicu, Thomas E. Nichols, for the Alzheimer's Disease Neuroimaging Initiative

**Affiliations:** ^1^ MRC Biostatistics Unit University of Cambridge Cambridge UK; ^2^ Geneva School of Economics and Management Research Institute for Statistics and Information Science, University of Geneva Geneva Switzerland; ^3^ Department of Statistics University of Warwick Coventry UK; ^4^ The Alan Turing Institute London UK; ^5^ Department of Statistics North Carolina State University Raleigh North Carolina USA; ^6^ Li Ka Shing Centre for Health Information and Discovery, Nuffield Department of Population Health Oxford Big Data Institute, University of Oxford Oxford UK; ^7^ Wellcome Centre for Integrative Neuroimaging, FMRIB, Nuffield Department of Clinical Neurosciences University of Oxford Oxford UK

## Abstract

Tensor‐based morphometry (TBM) aims at showing local differences in brain volumes with respect to a common template. TBM images are smooth, but they exhibit (especially in diseased groups) higher values in some brain regions called lateral ventricles. More specifically, our voxelwise analysis shows both a mean–variance relationship in these areas and evidence of spatially dependent skewness. We propose a model for three‐dimensional imaging data where mean, variance and skewness functions vary smoothly across brain locations. We model the voxelwise distributions as skew‐normal. We illustrate an interpolation‐based approach to obtain smooth parameter functions based on a subset of voxels. The effects of age and sex are estimated on a reference population of cognitively normal subjects from the Alzheimer's Disease Neuroimaging Initiative (ADNI) data set and mapped across the whole brain. The three parameter functions allow transforming each TBM image (in the reference population as well as in a test set) into a normative map based on Gaussian distributions. These subject‐specific normative maps are used to derive indices of deviation from a healthy condition to assess the individual risk of pathological degeneration.

## Graphical abstract

We propose a normative model trained on tensor‐based morphometry images from healthy individuals, which takes into account the asymmetric behaviour of the voxelwise distributions across brain locations. The model returns a new brain map for each image and indices of deviation from the healthy population.

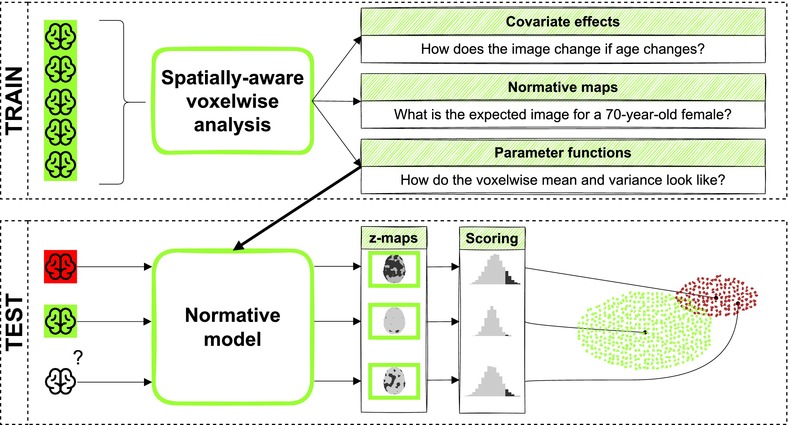

## 1 Introduction

The study of shapes and volumes of brain regions represents a valid approach to highlight differences between subjects (Ashburner and Friston 2004). Many phenomena, non‐pathological (like ageing) as well as pathological (e.g., Alzheimer's disease), are characterised by increasing atrophy at differential rates throughout the brain lobes. The atrophy is shown (cross‐sectionally between subjects or longitudinally) through the deformation of structural magnetic resonance images (sMRI).

Within the family of brain morphometry methods, tensor‐based morphometry (TBM) is used to identify regional volumetric deviations from a common sMRI template (Hua et al. 2013). The numeric value at each brain location can be interpreted as a multiplicative factor of expansion or shrinkage of the brain area. In particular, values above 1 in a brain area indicate that the subject shows an expanded volume with respect to the common template: for example, a TBM value of 1.1 means that the volume in the voxel of the subject image is 10% larger than the volume in the same voxel in the common template. For each voxel, this multiplicative factor of expansion/contraction is computed as the determinant of a Jacobian matrix which represents the alignment between the MRI image and the template (Ashburner and Friston 2004; Chung 2013). To model the distribution of TBM values, the log‐normal distribution has been considered (several arguments in favour of this option, including a better account of skewness, are listed in Leow et al. 2007). Nevertheless, the features of the voxelwise distributions have not been fully explored in the literature about TBM imaging. In particular, it is unclear also how those features vary between subjects in the healthy population and patients.

Normative modelling is a framework proposed in psychiatric and other clinical applications for studying inter‐individual variation with respect to a reference population (Rutherford et al. 2022). The relationship between an individual response variable and some clinically relevant covariates (e.g., age and sex) is estimated using a statistical model, which returns a description of the reference population in terms of quantiles, as in the growth charts used in several healthcare settings. The deviation from the centre of the reference population is quantified via a score (often based on the normal distribution, therefore known as *z*‐score or *z*‐value), which is used in subsequent analysis to return individual predictions based on the reference population (Marquand, Rezek, et al. 2016; Marquand et al. 2019). The *z*‐values effectively transform quantiles of the original distributions to the same quantiles of a standard normal distribution. With imaging data, a *z*‐value for each voxel can be computed so that each individual prediction is plotted as a so‐called *z‐map* (Ziegler et al. 2014; Marquand et al. 2019). The outcomes of the normative model (either the *z*‐maps or some numerical summaries of them) are often used for further analysis (e.g., classification tasks or other regression models).

Normative modelling is a suitable approach when there is no clear‐cut separation between the groups of healthy subjects and patients. For example, different subjects might show some aspects of the disease that could require the definition of subgroups of the disease or even a broader continuous spectrum of the pathology. In some diseases, it could also be argued that the disease cluster cannot be clearly separated from the healthy subpopulation (Marquand, Wolfers, et al. 2016). These views about the disease as a condition with large heterogeneity are not captured in the usual case–control approach, which is useful for comparing the averages in the two clinical groups but does not focus on the individual variation (which is often seen as a ‘residual’ under that framework). This approach is especially appealing in Alzheimer's disease studies, where there is a large interest in early detection of the disease. To this aim, Verdi et al. (2024) proposed a normative model based on structural MRI for Alzheimer's disease patients, using the count of outlying voxels as summary.

A growing body of literature has been recently published on normative modelling, focusing both on clinical application and methodological developments. Bethlehem et al. (2022) proposed a normative model where the evolution of seven brain phenotypes (dealing with tissue volumes and cortical summaries) across the human lifespan was modelled on a large aggregated sample of MRI images from multiple data sources. The brain charts were used to derive milestones in healthy brain development and to evaluate how ‘extreme’ the phenotypes of disease groups appeared with respect to the median of the normative population. A percentile score was used to quantify those differences, in a similar fashion as for the quantile rank maps proposed by Chen et al. (2015) in a study of functional connectivity. Several recent works have also focused on the improvement of normative models by replacing normal distributions with more flexible distributions, for example, generalised additive models for location, scale and shape (GAMLSS; Dinga et al. 2021), sinh‐arcsinh (SHASH) family and their reparametrisations (Fraza et al. 2021; Dinga et al. 2021; de Boer et al. 2024). In addition, the validation of normative models and evaluation of model performances and predictions has been extensively studied (Dinga et al. 2021; Ge et al. 2024).

In this work, we present a strategy for normative modelling of three‐dimensional (3D) TBM imaging data, which shows asymmetry in the voxelwise distributions (in other words, the distributions across subjects of TBM values observed for each voxel). The voxelwise distributions are modelled using skew‐normal distributions (Azzalini 2013) that offer a flexible extension of the normal distribution by means of an additional parameter (skewness). We also specify the mean parameter as a function of age and sex and use a convenient basis function specification, which allows us to exploit the spatial smoothing to avoid fitting a regression model per voxel.

**FIGURE 1:**
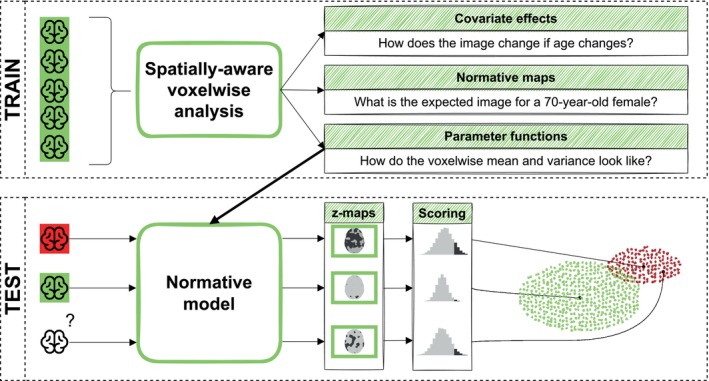
Scheme of the analysis. In the training phase, the parameters of the normative distribution are estimated for each voxel in a set of TBM images from healthy subjects (in green) and smoothed across the brain (spatially‐aware voxelwise analysis). The voxelwise analysis allows to derive sex‐specific, age‐adjusted mean and to predict the expected image for specific values of the covariates. In the test phase, the normative model (fully defined by the parameter functions in the training phase) is used to transform the brain images of subjects with a disease (in red) or yet undiagnosed (identified with question mark) into *z*‐maps which are referenced to the healthy population. For each subject, the values in the *z*‐maps are plotted in a histogram, and a score is defined (based on the values greater than a given threshold) to quantify the ‘extremity’ with respect to the median of the normative population.

The key goal of this work is to apply the normative modelling framework on a TBM data set from Alzheimer's Disease Neuroimaging Initiative (ADNI; Mueller et al. 2005), a large multi‐centre, observational study aimed at finding biomarkers for the early detection of Alzheimer's disease. For this goal, the quantification of atrophy both in healthy controls and patients is of great interest; therefore, our normative model proposal is aimed at identifying potential features linked to the disease. In particular, we want to describe the inter‐individual differences in regional brain volumes within the healthy reference population (top panel of Figure 1) and assess how far the TBM image of a subject (not included in the training set) is from the reference population (bottom panel of Figure 1). For this purpose, we consider some summaries of the *z*‐maps obtained from the normative model, capturing the extent of the departure of a subject from the normative population. In this way, we expect subjects with clinically measurable neurodegeneration to appear as extremes with respect to the normative population.

The normative TBM‐based *z*‐maps carry additional information with respect to the original images because they directly encode the relationship between the single image and the median brain volumes in the healthy population. This represents a great advantage in terms of the readability of the *z*‐maps, where values closer to 0 indicate brain volumes closer to the median pattern observed in the reference population. Indeed, observing, for example, a large TBM value does not indicate by itself whether the corresponding brain area has an ‘outlying’ expansion that raises suspicions of a disease, while through the *z*‐maps, this could be more easily assessed.

From a methodological standpoint, we also aim to introduce a more efficient approach to normative modelling by fitting the voxelwise distributions only on a subset of voxels and using spatial smoothing to obtain the full *z*‐maps. This approach requires the definition of a grid of voxels and an appropriate smoothing matrix, and it is particularly suitable for TBM images, which show some smoothness by construction (Hua et al. 2013).

The paper is structured as follows. We describe the features of the statistical model in Section 2, with a focus on voxelwise distributions and prediction for new subjects. We also illustrate the computational aspects related to the proposed interpolation approach, which avoids having to perform the estimation in all voxels while enforcing spatial smoothness. In Section 3, we describe the TBM data set used for the analysis and display the main features of interest (namely, a mean–variance relationship and skewness at the voxelwise level), which motivate our modelling choice. The results for the normative population are shown along with an example of *z*‐map scoring by diagnosis group. Finally, we discuss potential further developments of the model in Section 4.

## 2 Methods

### 2.1 Statistical Framework

Let $Y_{i}=\left\{Y_{i}\left(v\right),v\in V\right\}$ be the brain image for the i th subject (i=1,…,N), whose domain is the closed cube $V\in {\mathbb{R}}^{3}$. We assume that $Y_{i}$ is a square‐integrable random function on V. In practice the domain V is discretised into V voxels $v_{1},\ldots ,v_{V}$; therefore, in the next sections, we will refer to the observed data for the i th subject as $Y_{i}\left(v_{j}\right),\;j=1,\ldots ,V$.

For v∈V, define

(1) $$U_{i}\left(v\right)=F_{\mathrm{SN}}\left(Y_{i}\left(v\right);\mu \left(v\right),\sigma^{2}\left(v\right),\gamma \left(v\right)\right),$$

where $F_{\mathrm{SN}}$ denotes the skew‐normal cumulative distribution function in its so‐called *centred parameterisation* (CP; see Azzalini 2013; Arellano‐Valle and Azzalini 2008; we briefly describe the probability distribution function in Supporting Information S1). In this CP form, we refer to the parameters as mean function μ, standard deviation function σ and skewness function γ for the reference population. The set of parameters of $F_{\mathrm{SN}}$ is called CP to distinguish it from the original direct parameterisation (DP). The likelihood function for CP gets closer to a quadratic function and produces estimators which are less correlated than the DP estimators (Monti 2003).

As noted in Azzalini (2013), the skewness parameter γ is constrained within the set $\left(-c_{\gamma },c_{\gamma }\right)$, with

(2) $$c_{\gamma }=\frac{\sqrt{2}\left(4-\pi \right)}{{\left(\pi -2\right)}^{3/2}}\approx 0.9953,$$

while other distributions such as skew‐t might be more appropriate for larger observed sample skewness. The direction of the skewness is determined by the sign of $\gamma \left(v\right)$: if positive, the distribution is skewed to the right. For skewness equal to 0, SN reduces to a normal distribution with the same mean and variance.

The probability integral transform in Equation (1) $U_{i}\left(v\right)\in \left(0,1\right),\;\forall v\in V$: it is a latent uniform process based on the distribution $F_{\mathrm{SN}}$ for the ith subject. In other words, this returns the quantiles for the distribution in the reference population. Covariates can be easily accommodated in this framework. For example, a linear effect of the covariates X on the mean parameter can be included as $\mu \left(v\right)=X\beta \left(v\right),\;\forall v\in V$ (see Li et al. 2015 for a more general formulation). In this work, we assume a linear model for the mean of the skew‐normal distribution, with the design matrix X is made of four columns: a vector of 1 for the intercept and the values of age, sex and their interaction. The sn package allows for the use of covariates only in the location parameter (Azzalini 2013), using an iterative procedure for the maximisation of the likelihood function in the CP form.

The latent process $U_{i}$ has the role of incorporating the voxelwise distributional differences in a single object. At the voxelwise level, its interpretation as quantile of a distribution returns an immediate quantification of the distance from the median of the population (identified as $U_{i}\left(v\right)=0.5\;\forall v\in V$). The values of $U_{i}$ are therefore comparable with each other, in contrast to the original TBM values: indeed, while a TBM value of 1100 might be totally within a normal range in one brain location and extreme in another, a $U_{i}\left(v\right)$ value of 0.7 has the same interpretation across the whole brain.

Let $Y^{*}$ be a realisation of a random function for a new subject who could either belong or not to the reference population. We can compute the latent uniform process $U^{*}\left(v\right)$ using Equation (1) with the estimated parameters obtained in the reference population, and set

(3) $$Z^{*}\left(v\right)=\Phi^{-1}\left(U^{*}\left(v\right)\right).$$

where $\Phi^{-1}$ is the inverse cumulative distribution function of a standard normal. In this way, the quantiles of the original voxelwise distribution are mapped to the quantiles of a standard normal distribution. This normative *z*‐map gives information about how the image for the new subject compares to the reference population: values closer to 0 indicate that the volume observed locally in the image for the subject is close to the median value in the reference population, whereas more extreme values are potentially informative of non‐healthy expansion/shrinkage of brain regions. The degree of information of the *z*‐map is also more homogeneous across the brain than the original TBM values: while a value of 3 in the *z*‐map indicates the same degree of extremity for all the voxels, a value of 1100 in a TBM image might be within a normative range in one brain area but not in another. The *z*‐map for each subject could be therefore used to immediately locate those brain areas that depart from the mean of the normative population.

Various scalar indices can be built to summarise the information carried by the *z*‐maps into a single value. A simple approach is to plot first the *z*‐values for each subject into a histogram and then take a summary statistics of the distributions or parts of it, like the tails. For example, each individual *z*‐map can be summarised by the (robust) mean of an extreme tail, from the 99th percentile of the distribution of *z*‐values for each subject (other indices based on extreme value analysis are presented in Marquand, Rezek, et al. 2016). Under this approach, the specific application will drive the choice of the percentile defining the extreme tail (whether to deal with the highest, lowest or both). In our setting, where the enlargement of the ventricles is balanced by the shrinkage of the cortex, it seems likely that extremes in both sides are carrying information; therefore, we will define the summary $u_{q}^{\mathrm{abs}},\;q\in \left(0,1\right)$ as the mean of the right tail above quantile q of the distribution of absolute *z*‐values.

### 2.2 Model Estimation

In imaging data, normative modelling is performed at each voxel independently, without directly including the smoothness of the images in the model. This approach is not ideal for two important reasons. The first reason is computational: although parallelisable, the calculation of the parameters at the voxelwise level will still require some time, since the number of voxels is large. The second reason is more conceptual: the full voxelwise approach will not ensure the smoothness of the functional parameter by default (which is a desirable outcome, because the noise induced by the discretisation is not of interest and because the storage in memory of the smoothing basis expansion is more efficient). In other words, neighbouring voxels often carry a similar amount of information, which could be more efficiently represented if we introduce some degree of smoothness. Smoothness of brain images has also been studied in the normative modelling literature (Ziegler et al. 2014).

**FIGURE 2:**
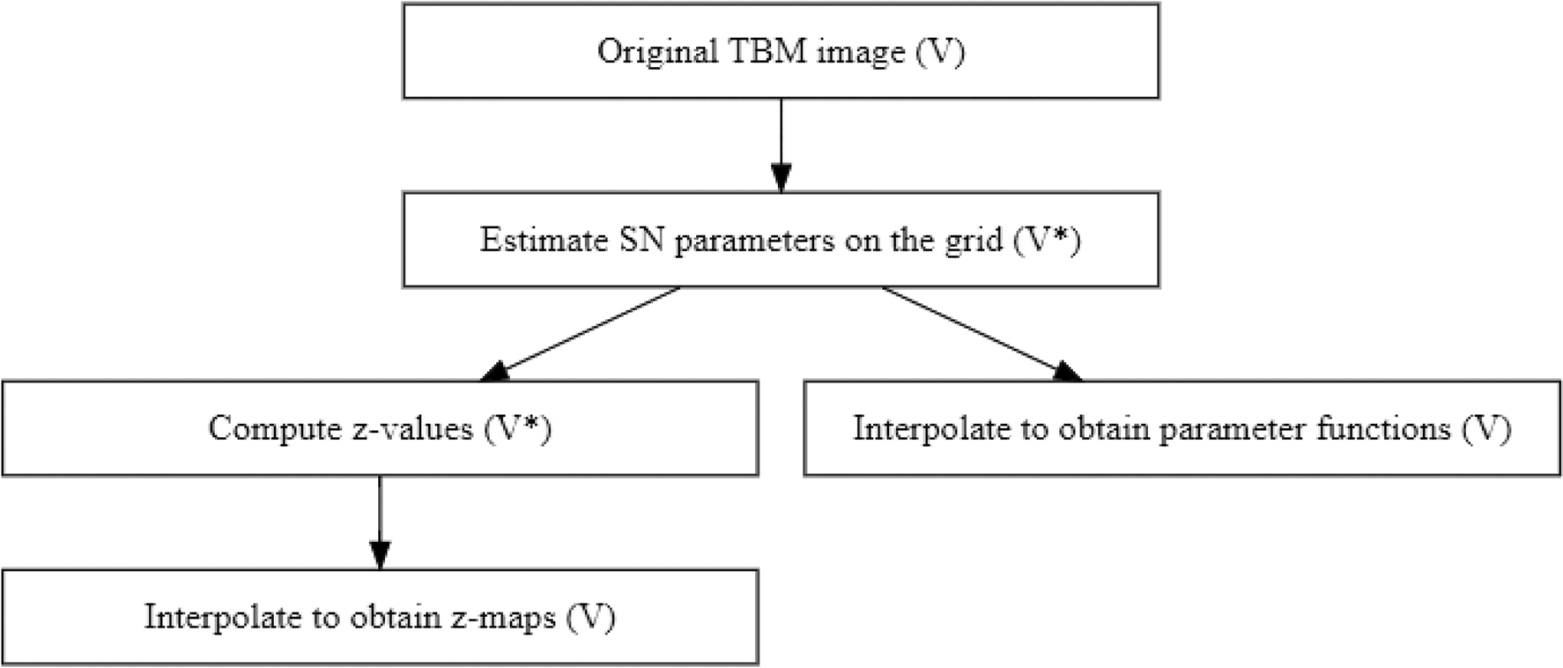
Workflow for the analysis on the grid (the number of voxels considered in the corresponding step is reported in brackets). Starting from the original TBM image, for each voxel in the grid, the parameters of the skew‐normal distribution are estimated (and interpolated across the whole brain to obtain the parameter functions). Using those parameters, the *z*‐values are computed on the grid. Radial basis functions (RBFs) are then used to estimate the *z*‐values in the rest of the brain image. Once the full *z*‐maps are calculated for each subject, these can be used for further analysis (e.g., performing functional principal component analysis to identify the main modes of variation.

To bypass the need of running the computation for every voxel, we consider a subset of the voxels arranged in a regular grid K of size ($V^{*}\ll V$). From now on, those selected voxels will be defined as $\left\{\kappa_{1},\ldots ,\kappa_{V^{*}}\right\}$. For these voxels, the SN regression models are fitted, and the maximum likelihood estimates of the parameters are computed. For the ith subject in the reference sample, the z‐values can be computed as in Equations (1) and (3). The analysis based on the grid (summarised in Figure 2) is detailed below.

For the interpolation of the *z*‐values for voxels outside the set K, we use radial basis functions (RBFs; Fasshauer and Zhang 2007). The number of RBFs is determined by the number of ‘centres’, that is, fixed points at which the radial function takes their largest value. A basis function for each centre is computed, with non‐zero weights in the voxels that are within a certain radius of the centre. The standard choice for centres is the same grid of preselected voxels ${\left\{\kappa \right\}}_{j=1}^{V^{*}}$ where we have carried out the likelihood maximisation. We recall that the observed value of the function at the jth centre is $Z\left(\kappa_{j}\right)$.

Following the mathematical procedure illustrated in Carr et al. (2001), we define the interpolant s as a real‐valued function with constraints $s^{*}\left(\kappa_{j}\right)=Z\left(\kappa_{j}\right)$ (i.e., the observed value of the function at the jth centre). To define $s^{*}$, we build the matrix

(4) $$G=\left(\begin{array}{cc} H^{*} & 1\\ 1^{T} & 0 \end{array}\right)$$

where the $V^{*}\times V^{*}$ symmetric matrix $H^{*}$ contains the evaluation of the RBF h for any distance d between any pair of centres

(5) $$H_{\mathrm{jl}}^{*}=h\left(d\left(\kappa_{j},\kappa_{l}\right)\right)\;j,l=1,\ldots ,V^{*}.$$

and 1 is the $V^{*}$‐dimensional vector whose elements are equal to 1. The problem is now phrased in terms of a linear system: we are interested in finding the $V^{*}$‐dimensional vector b and the scalar $b_{0}$ such that

(6) $$Z\left(\kappa_{j}\right)=b_{0}+\sum_{l=1}^{V^{*}}\;b_{l}H_{\mathrm{jl}}^{*}.$$

with a sum‐to‐one constraint on the vector b. The solution is now used to predict a value for a generic voxel:

(7) $$\left(H\;1\right)\left[\begin{array}{c} b\\ b_{0} \end{array}\right]$$

where H is the V×V matrix with the RBF evaluated at each voxel.

The gain in computational efficiency that stems from applying basis functions on the grid instead of using all the voxels in the brain comes at a price. First, the performance of smoothing basis functions relies on some tuning parameters (such as the standard deviation for Gaussian RBF), which makes not easy to determine optimality criteria. The best parameter is often chosen by trial and error or ad hoc solutions (Fasshauer and Zhang 2007). Indeed, when the basis covers a larger area, the interpolation matrix becomes less sparse, while in the opposite case, the so‐called ‘bed‐of‐nails’ interpolant is obtained: the function sharply peaks at the centres but decreases to 0 elsewhere. In the 3D grid case, we suggest selecting a value for the tuning parameter that is smaller than the distance between consecutive centres in the grid. In this way, the fitted values on the grid are not changed, and only the voxels outside the grid are interpolated.

Another aspect of interpolation using RBF and polynomials is Runge's phenomenon, that is, the approximation errors further from the centres are larger at the boundary of the domain (Fasshauer and Zhang 2007; Boyd 2010). In the 3D brain imaging setting, although the brain mask has irregular boundaries in the three dimensions, this issue is not likely to be relevant, especially when the grid spacing (and consequently the maximum distance between a voxel and the closest centre) is moderate.

In practice, the interpolation procedure requires the choice of a grid of voxels and the construction of the interpolation matrix. The interpolation is then applied to the parameter functions (including the age and sex coefficients), as well as the *z*‐maps. The code for the spatial interpolation, as well as for the results in the next section, is available at https://github.com/marcopalma3/NormMap3D.

## 3 Results

### 3.1 Data

**FIGURE 3:**
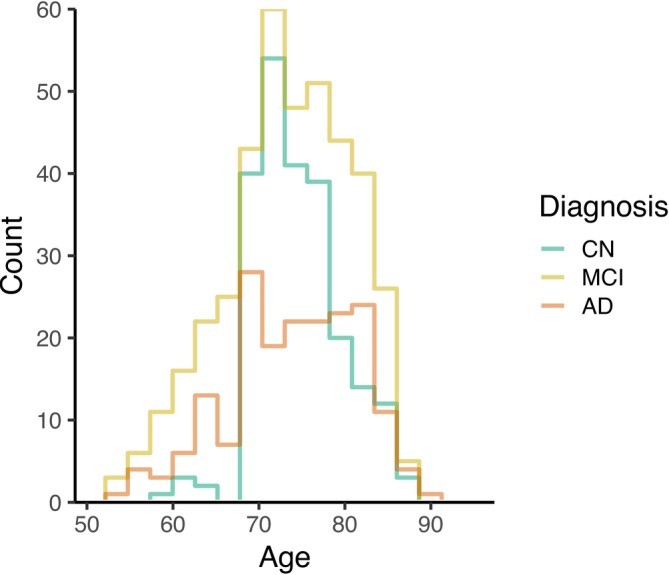
Histogram of age by diagnosis group in the TBM ADNI sample.

We build the normative model on a data set from ADNI, which consists of 817 adults (with age ranging between 54.4 and 90.9 years). A diagnosis is available for each of them (Table 1): 229 subjects were considered as cognitively normal (CN), whereas 400 subjects were showing mild cognitive impairment (MCI), and 188 were diagnosed with Alzheimer's disease. The histogram of age by diagnosis group is shown in Figure 3.

**TABLE 1:** Demographic characteristics of the subjects in the data set.

Diagnosis	Subjects	Proportion of females	Age mean	Age IQR
CN	229	0.48	75.87	6.20
MCI	400	0.36	74.74	10.23
AD	188	0.47	75.36	10.58

Abbreviation: IQR, interquartile range.

The imaging data used in this work are TBM images. In a cross‐sectional setting, each MRI scan is aligned to the *minimal deformation template* (MDT) obtained by averaging several structural MRI scans (Hua et al. 2013). The deformation induced by this alignment is mathematically described by a function that maps a 3D point in the template to the corresponding one in the individual image. The Jacobian matrix of the deformation incorporates the volume differences with respect to the MDT in terms of shearing, stretching and rotation. Its determinant evaluated at each voxel is a summary of local relative volumes compared to the MDT. Further details about TBM data are available in Ashburner and Friston (2004).

A 3D preprocessed TBM image taken at the entrance of the ADNI study is available for each individual in the sample. The dimensions of the images are 220×220×220, with voxel size equal to 1 mm^3^. In the data set, the threshold value is set at 1000: larger values indicate that expanded volume with respect to the MDT is observed in that specific voxel (lower values indicate shrinkage). Information about the preprocessing stages for the ADNI TBM data set is available at https://adni.bitbucket.io/reference/docs/TBM/ADNI_Methods_TBM_IGC_LONI_Oct2012.pdf.

The mask used to subset only the part of the image that displays the brain is built with the same characteristics as described in Palma et al. (2020): we use a Gaussian kernel with a standard deviation equal to two voxels (FWHM 4.7 mm) and threshold it at 0.5. Each masked image is made up of approximately 2 million non‐zero voxels.

### 3.2 Exploratory Analysis

**FIGURE 4:**
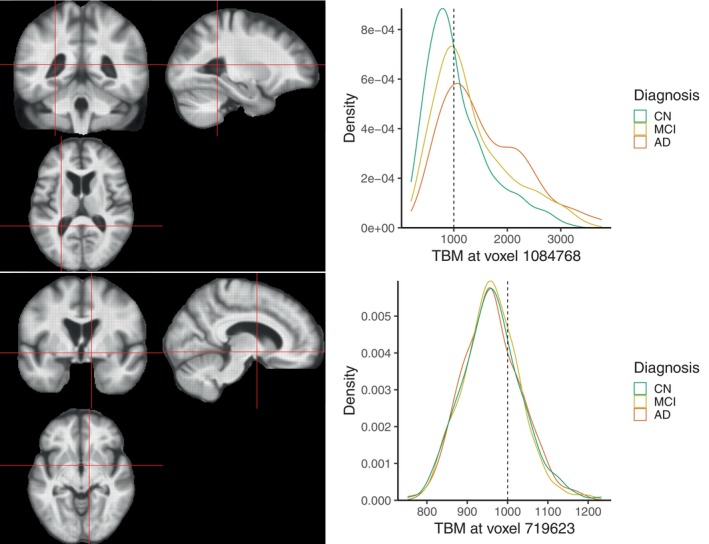
Top: Empirical density functions of TBM Jacobian values by diagnosis group (right) for a voxel in the lateral ventricles. Bottom: Empirical density functions of TBM Jacobian values by diagnosis group (right) for a voxel outside the lateral ventricles.

The exploratory analysis of the TBM images reveals spatial patterns in the voxelwise distributions. Figure 4 shows the estimated densities of TBM values by diagnosis groups for two example voxels in the brain, located inside and outside the lateral ventricles, respectively. The patterns observed are very different: for the voxel in the ventricles, all densities within the ventricles show a longer right tail, which is heavier for the AD group compared to MCI and CN, while the voxel outside the ventricles shows no clear differences between the densities across the groups.

**FIGURE 5:**
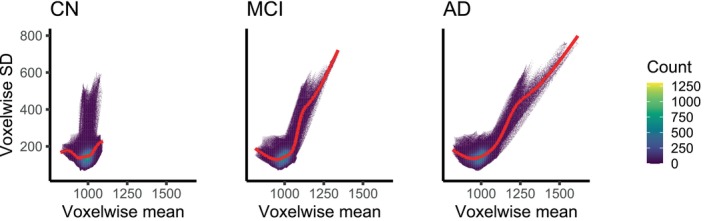
2D histograms of voxelwise means and standard deviations (SDs) by diagnosis group. The number of bins is fixed to 600. A smooth regression line (obtained via penalised cubic splines) is added in red.

**FIGURE 6:**
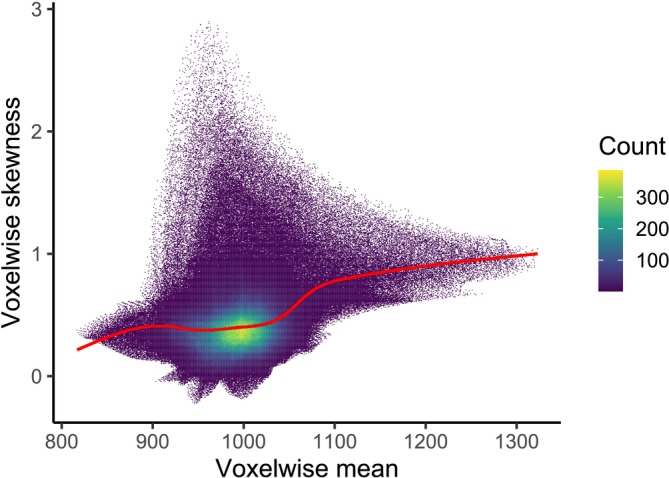
2D histogram of the voxelwise mean and skewness across all subjects. The number of bins is fixed to 600. A smooth regression line (obtained via penalised cubic splines) is added in red.

When looking at the summary statistics for all the voxels across all subjects (without introducing the covariates), the patterns between diagnosis groups are even more evident. Figure 5 shows the relationship between voxelwise means and standard deviations for each group. While most voxels show a mean around 1000, larger means are observed for some voxels, especially for the groups with diseases. But even for the CN subjects, the standard deviations change as the means increase. When we compute the Pearson's moment coefficient of skewness for each voxel (Figure 6), brain areas with larger means (e.g., the lateral ventricles) tend to exhibit more skewed distributions.

### 3.3 Mapping the CN Population

We consider first the normative sample (training set) to compute the skew‐normal parameter estimates: we split the CN group in five folds, four of which were used for training (as done, e.g., in Rutherford et al. 2022), with stratification by age group and sex (for a total of 183 individuals, of which 95 males and 88 females). We carry out the skew‐normal fitting procedure on a regular grid with 8 mm spacing in the three dimensions. This returns 3949 voxels, approximately equal to 0.2% of all the voxels within the mask. For these voxels, the skew‐normal regression models (with CP) are fitted using the R package sn (Azzalini 2020). Only the mean is modelled as a function of age, sex and their interaction.

RBFs with Gaussian kernel and standard deviation ε=5.33 mm (66.67% of the grid spacing) are used to interpolate the SN parameter functions across the rest of the brains. This value represented a compromise to achieve good interpolation quality while avoiding the bed‐of‐nails issue.

**FIGURE 7:**
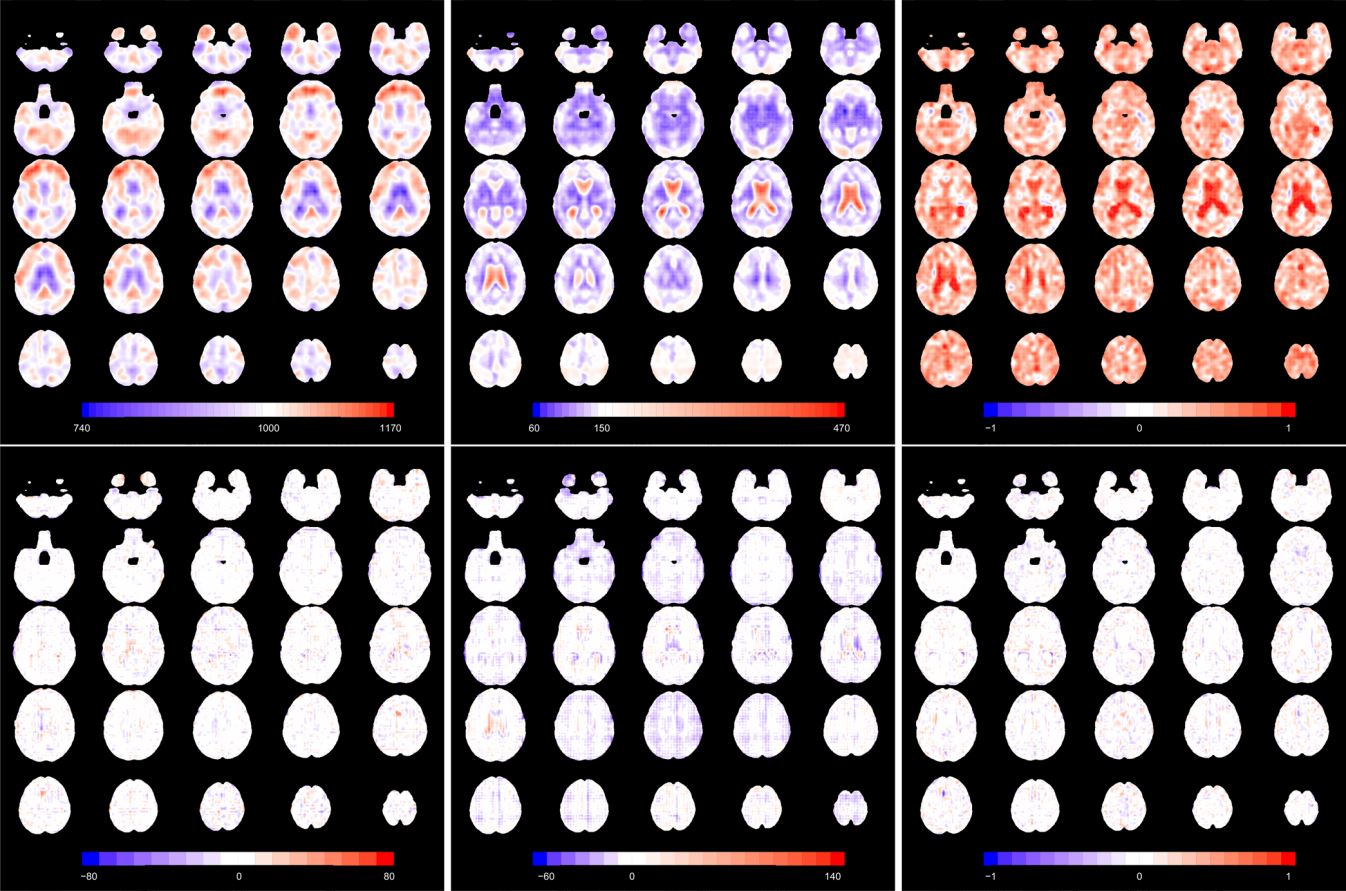
Axial slices of the intercept (left), standard deviation (centre) and skewness (right) parameter functions from skew‐normal fitting in the normative population. Slices are ordered from bottom to top. Top plots refer to the grid interpolation approach, while bottom plots are the difference between the full fit and the interpolation approach.

As a validation of this grid fitting procedure, we also compared the estimated parameters with the full version, where a skew‐normal regression model is fitted independently at each voxel. While the grid approach takes approximately 30 s in a serial version, the full approach (parallelised on 10 cores) takes approximately 2.5 h on a standard laptop. We show the estimated parameter functions for the interpolation approach in Figure 7, as well as the differences with the full fit approach. The difference between the full and interpolated version is very small for all parameters, except for the standard deviation within the ventricles, which is larger in the full version. In general, the mean and the standard deviation are larger in the lateral ventricles than in the rest of the brain. The estimated skewness is positive across almost the entire mask.

**FIGURE 8:**
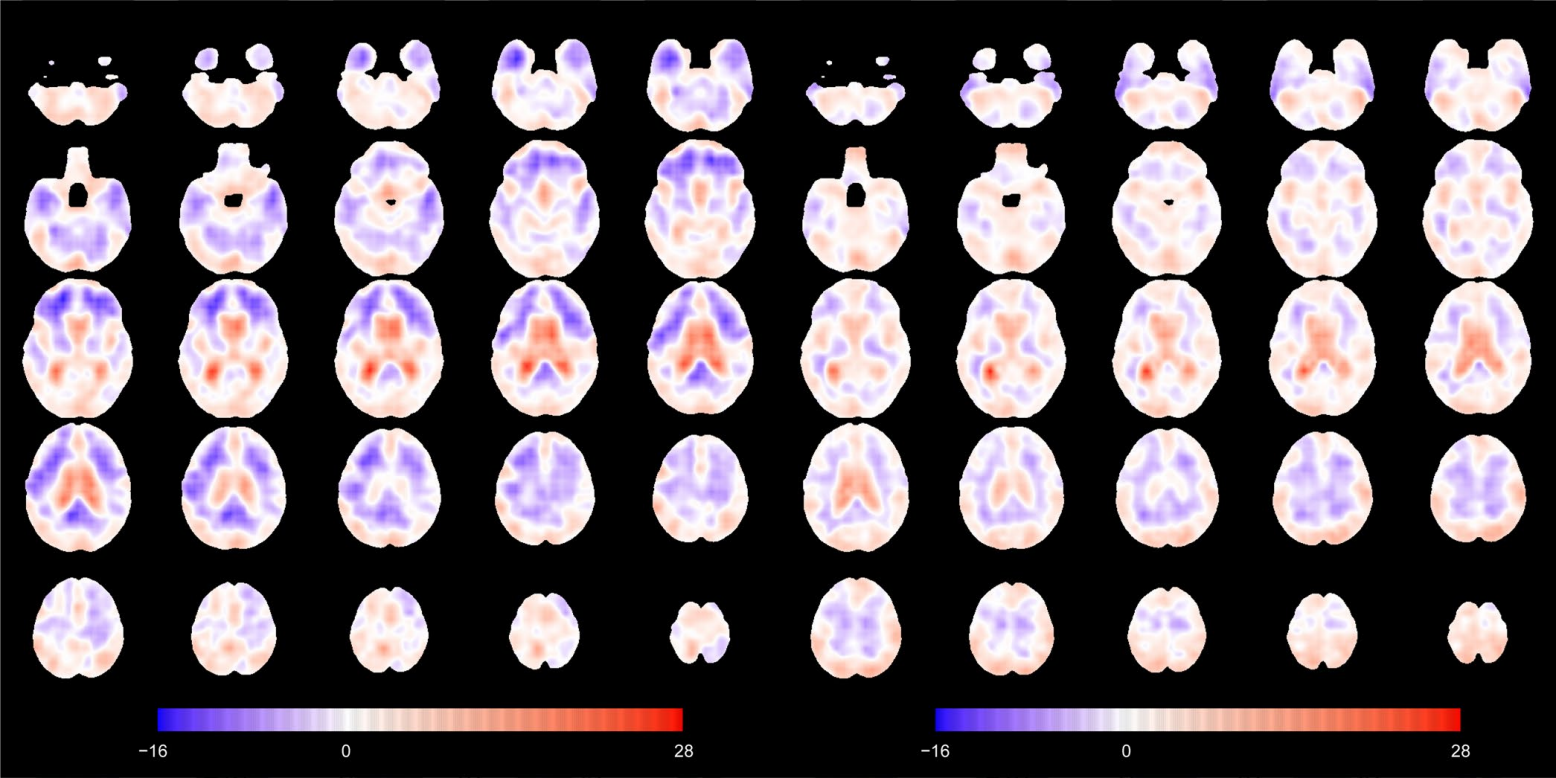
Axial slices of the age association with the mean function for females (left) and males (right) in the normative population. Slices are ordered from bottom to top.

For the mean of the normative population, we also display the linear effects of age and sex (and their interaction) on the TBM maps (Figure 8). A 1‐year change in age for females is also linked to an expansion in the lateral ventricles, balanced by the shrinkage in other parts of the brain, particularly in the frontal lobe. The same 1‐year change in age for males mostly affects the same brain regions, although the volume differences are more modest.

These parameter functions are used to obtain mean predictions for specific covariate values in the normative population. For example, the mean for females at 70, 80 and 90 years is displayed in Supporting Information S1. While at 70 years old, the lateral ventricles are smaller than the TBM template, their volume becomes bigger than the template at 80 and increases at 90 years old. An opposite trend is observed for the volume in the frontal regions. In terms of the intensity of the TBM values, more extreme values are observed at the highest age level.

**FIGURE 9:**
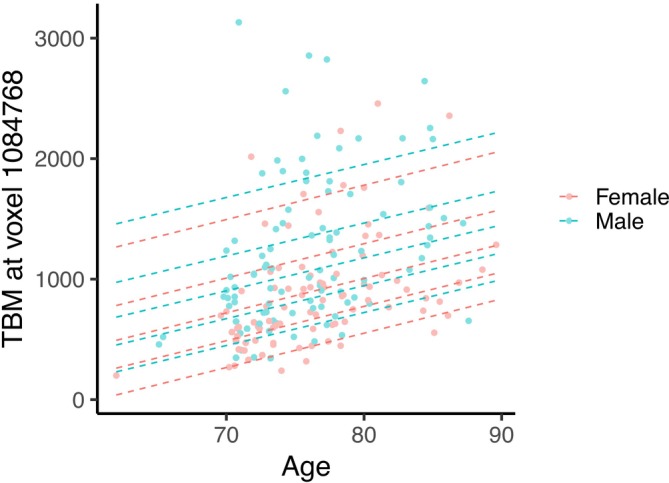
Scatterplot of TBM values by age for a voxel within the ventricles, with quantile lines as estimated from the normative model. The lines refer to quantiles 0.1, 0.3, 0.5, 0.7 and 0.9.

We also looked at the percentile lines (Figure 9) estimated from the model for the voxel within the ventricles shown in Figure 4. The TBM values tend to increase with age (as expected) and the skew‐normal model provides an indication of the skewness in the data (as shown by the increasing space between consecutive quantiles). The estimated coefficient for the corresponding skew‐normal model is reported in the table in Supporting Information S1, along with the ordinary least squares fit for the same model under a normal distribution. For this voxel, as skewness is large, the estimates between the two models are different, while for the voxel outside the ventricles, the estimates largely agree. This confirms that, when the distribution shows little skewness, the skew‐normal model is able to handle effectively the settings in which a symmetric normal distribution would be appropriate.

**FIGURE 10:**
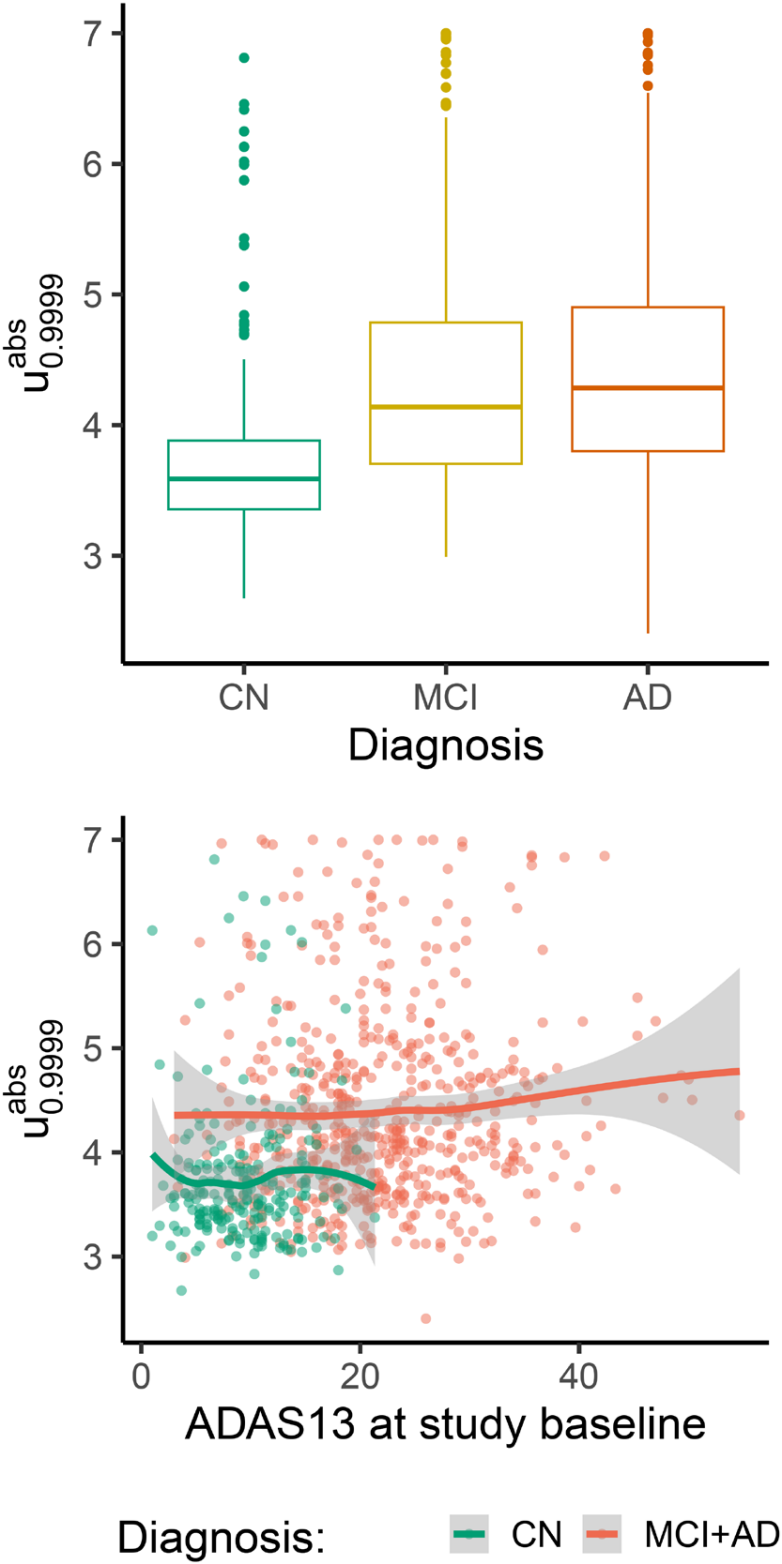
Top: Boxplots of $u_{0.9999}^{\mathrm{abs}}$ by diagnosis group. Bottom: Plot of $u_{0.9999}^{\mathrm{abs}}$ by ADAS13 and diagnosis group.

The *z*‐maps computed using the skew‐normal parameter values at the grid and then smoothed across the rest of the brain are obtained for both the subjects in the normative population and the test sets. Figure 10 (top) shows the boxplots of $u_{0.9999}^{\mathrm{abs}}$, the index of deviation obtained by averaging the top 0.01% *z*‐values in absolute values for each subject. Some evidence of a trend between this index and the severity of disease status is observed, although no relevant discrimination across groups is observed. In addition, when the index of deviation is plotted against ADAS13 (Kueper et al. 2018), a neuropsychological test often used in AD studies to assess cognitive dysfunctions (the larger the score, the higher the disease severity), the regression lines for the diseased groups remain above the one for CN subjects (Figure 10, bottom). Similar results (not reported here) were obtained for different thresholds of the right tail of the distribution.

## 4 Conclusions and Further Developments

The analysis of brain morphometry images is of great interest due to its ability to show and quantify signs of atrophy within different brain regions. Using a data set from ADNI, we have shown that the mean, standard deviation, and skewness of the voxelwise distributions of TBM values exhibit interesting spatial patterns. In this work, we have modelled these characteristics by using a skew‐normal distribution at the voxel level within a normative model to study brain volumes in the absence of neurodegeneration. The normative approach provides then a set of reference parameters on which to build individual brain maps, which can then be summarised into single indices. The normative modelling approach allows us to identify subjects with cognitive impairment as ‘extremes’ with respect to the reference population by means of individual risk scores.

A novel outcome of this normative model for TBM images is the quantification and visualisation of volume differences across brain regions within the healthy population. In particular, the standard deviation parameter function shows that even in the CN group, the voxel with the highest variability is located within the lateral ventricles. This atlas of TBM variability could be useful to better understand age‐related effects in regional volume differences and help disentangle pathological degeneration from the normal age effect. Following the normative modelling framework, once we have estimated the parameter functions, we can reference a test image to the normative population.

A novel contribution of this work is the illustration of a computationally efficient strategy to fit the regression models on a subset of voxels. This approach gives a substantial computational benefit even in the small training set (< 200 images) considered in this application. We expect interpolation‐based strategies to be even more beneficial for biobank‐scale data sets or for more computationally intensive models (e.g., Bayesian regression and permutation models).

A strength of this approach is the large flexibility given by the modular structure of the normative modelling workflow. The spatial interpolation procedure described in this paper is independent of the voxelwise distribution chosen, as well as from the score used to summarise the *z*‐maps; therefore, the data analyst can combine these elements with their distribution or score preferences. In this analysis, a single distribution family (the skew‐normal) was chosen as it provided enough flexibility to deal with the spatial patterns between voxels within and outside the lateral ventricles. Nevertheless, other continuous distributions (such as the ones in the GAMLSS framework, Rigby and Stasinopoulos 2005; Dinga et al. 2021) could be explored, especially for the ventricular regions, where the skewness bounds of the skew‐normal distribution could be restrictive. Information criteria such as AIC and BIC (as well as scoring rules computed on an independent validation set) can inform the choice between multiple candidate distributions (Bethlehem et al. 2022; Dinga et al. 2021; Rutherford et al. 2022). The indices of deviations can also be tailored for specific interests (e.g., considering only positive or negative extremes). In addition, other quantities like functional data depth (López‐Pintado and Romo 2009; Mosler and Polyakova 2012; Gijbels and Nagy 2017) could be extended to the case of 3D imaging data and applied in this context.

There are also some limitations to this approach. The voxelwise distribution considered in this work is quite restricted, as we have considered only two covariates (age and sex) in a linear model for the mean only. A flexible functional form for the mean, as well as the introduction of covariates in the other parameter functions, could be investigated to evaluate more complicated age‐related patterns. In addition, the grid computation needs to be carefully checked, as the loss in the smoothing step can increase for a poor choice of smoothing parameters (for which an automatic selection criterion is not easy to determine). In addition, different smoothing levels for the distributional parameters, as well as the sensitivity to the choice of the voxels in the grid, could be further explored (e.g., in our setting, we observe that the interpolation is particularly good for the mean, less so for the standard deviation function).

In addition, the relatively limited size of the training set in this normative model makes the evaluation of outlying percentiles and the estimation at the boundaries of the age domains less reliable (Bozek et al. 2023), in particular for individuals younger than 70 years In particular, the accuracy of the estimation of those percentiles could be further reduced by the interpolation step. Further validation of the impact of interpolation, as well as replication of the entire workflow over a larger TBM data set, would be useful to confirm the results shown here and assess model uncertainty.

The normative model workflow illustrated here might also be used as a preliminary step for further analysis based on a normative population. Normative *z*‐maps could be used, for example, within a regression framework to predict the disease status or transition from CN to AD. For these tasks, scalar‐on‐function regression (Morris 2015) employs the whole image as the independent variable, for example, through its basis representation or the scores obtained from a functional principal component analysis (Ramsay and Silverman 2005). In addition to the voxelwise marginal distributions, the spatial dependence could be modelled using a copula (as illustrated in Staicu et al. 2012 and extended in Li et al. 2015)—although the high dimensionality of the images would probably require some approximations. An interesting extension would also be represented by a longitudinal normative model based on multiple brain images over time, to better capture volume changes and quantify the risk of conversion to Alzheimer's disease.

## Author Contributions

Conceptualisation: all authors. Data acquisition: M.P. and T.E.N. Formal analysis and visualisation: M.P. Writing – original draft: M.P. Writing – review and editing: all authors.

## Conflicts of Interest

Thomas E. Nichols is a member of the HBM Editorial Board and co‐author of this article. The other authors declare no conflicts of interest.

## Supporting information


**Data S1.** Supporting Information.

## Data Availability

Data are available under acceptance of the Data Use Agreement through the LONI Image and Data Archive (IDA). Data collection and sharing for this project was funded by the Alzheimer's Disease Neuroimaging Initiative (ADNI) (National Institutes of Health Grant U01 AG024904) and DOD ADNI (Department of Defense award number W81XWH‐12‐2‐0012). ADNI is funded by the National Institute on Aging, the National Institute of Biomedical Imaging and Bioengineering, and through generous contributions from the following: AbbVie, Alzheimer's Association; Alzheimer's Drug Discovery Foundation; Araclon Biotech; BioClinica Inc.; Biogen; Bristol‐Myers Squibb Company; CereSpir Inc.; Cogstate; Eisai Inc.; Elan Pharmaceuticals Inc.; Eli Lilly and Company; EuroImmun; F. Hoffmann‐La Roche Ltd. and its affiliated company Genentech Inc.; Fujirebio; GE Healthcare; IXICO Ltd.; Janssen Alzheimer Immunotherapy Research & Development LLC.; Johnson & Johnson Pharmaceutical Research & Development LLC.; Lumosity; Lundbeck; Merck & Co. Inc.; Meso Scale Diagnostics LLC.; NeuroRx Research; Neurotrack Technologies; Novartis Pharmaceuticals Corporation; Pfizer Inc.; Piramal Imaging; Servier; Takeda Pharmaceutical Company; and Transition Therapeutics. The Canadian Institutes of Health Research is providing funds to support ADNI clinical sites in Canada. Private sector contributions are facilitated by the Foundation for the National Institutes of Health (www.fnih.org). The grantee organisation is the Northern California Institute for Research and Education, and the study is coordinated by the Alzheimer's Therapeutic Research Institute at the University of Southern California. ADNI data are disseminated by the Laboratory for Neuro Imaging at the University of Southern California.
