# Supplementary material for: Smooth Normative Brain Mapping of Three‐Dimensional Morphometry Imaging Data Using Skew‐Normal Regression
Source: Hum Brain Mapp. 2025 Mar 3;46(4):e70185. doi: 10.1002/hbm.70185 (PMC11875072; doi:10.1002/hbm.70185)
Supplement: Supplementary file 1 — Data S1. Supporting Information. [file HBM-46-e70185-s001.pdf]

Supplementary Material

Smooth normative brain mapping of 3-dimensional morphometry imaging data using skew-normal regression

1 Skew-normal distribution

The description of the skew-normal distribution provided here is just an introduction to the topic, which is better described in Arellano-Valle and Azzalini (2008) and Azzalini and Capitanio (2014).

The probability density function of a skew normal random variable X defined over the support $(-\infty, \infty)$ is

$$f(x) = 2\phi(x)\Phi(\alpha x) \tag{1}$$

where $\phi(x)$ is the density of a standard normal and $\Phi(\alpha x)$ is the distribution function of a standard normal evaluated at αx. The parameter α controls the shape of the skew-normal density function: for $\alpha = 0$, the skew-normal reduces to a standard normal, while for positive values of α a right-skewed distribution is obtained.

Consider now the linear transformation $Y = \xi + \omega X$, with location parameter ξ and scale parameter ω. The random variable $Y \sim SN(\xi, \omega^2, \alpha)$, with probability density function

$$f(y) = \frac{2}{w}\phi\left(\frac{x-\xi}{\omega}\right)\Phi\left(\frac{\alpha(x-\xi)}{\omega}\right). \tag{2}$$

This parameterisation is called *direct parameterisation* (DP). The expectation $\mathbb{E}(Y) = \mu$ and the variance $\mathbb{V}(Y) = \sigma^2$ are functions of the three parameters.

Let us now consider the variable $X_0 = \frac{X-\mu_X}{\sigma_X}$, where $\mathbb{E}(X) = \mu_X$ and the variance $\mathbb{V}(X) = \sigma_X^2$. The variable X_0 is now centred at zero. For the properties of transformation of random variables,

$$X \sim SN(0, 1, \alpha) \Rightarrow X_0 \sim SN\left(-\frac{\mu_X}{\sigma_X}, \frac{1}{\sigma_X}, \alpha\right).$$

We can now use the linear transformation $Y = \mu + \sigma X_0$ and specify the centred parameterisation $\theta^{(CP)} = (\mu, \sigma^2, \gamma_1)$ where γ_1 is a measure of skewness bounded within $(0.9953, 0.9953)$. The components of CP are functions of the first three moments and a mapping exists between DP and CP.

2 Additional results

	Voxel 1084768		Voxel 719623	
Coefficients	OLS	SN	OLS	SN
Intercept	623.848 (90.233)	812.012 (67.680)	946.698 (13.113)	946.775 (12.960)
Age	39.775 (10.959)	28.488 (6.960)	3.383 (1.593)	3.160 (1.576)
Sex(Male)	324.6 37 (123.334)	182.926 (78.468)	13.183 (17.923)	14.678 (17.658)
Age::Sex(Male)	5.197 (15.490)	-1.153 (10.182)	-3.286 (2.251)	-3.081 (2.211)
Residual SD	512.591	489.794	74.491	73.611
γ (skewness)	-	0.914 (0.053)	-	0.249 (0.171)

Table 1: Estimated coefficients for ordinary least squares (normal) and skew-normal regression models, for voxels 1084768 (within the ventricles) and 719623 (outside the ventricles).

References

Arellano-Valle, R. B. and Azzalini, A. (2008). The centred parametrization for the multivariate skew-normal distribution. *Journal of multivariate analysis*, 99(7):1362–1382.

Azzalini, A. and Capitanio, A. (2014). *The skew-normal and related families*. Cambridge University Press.

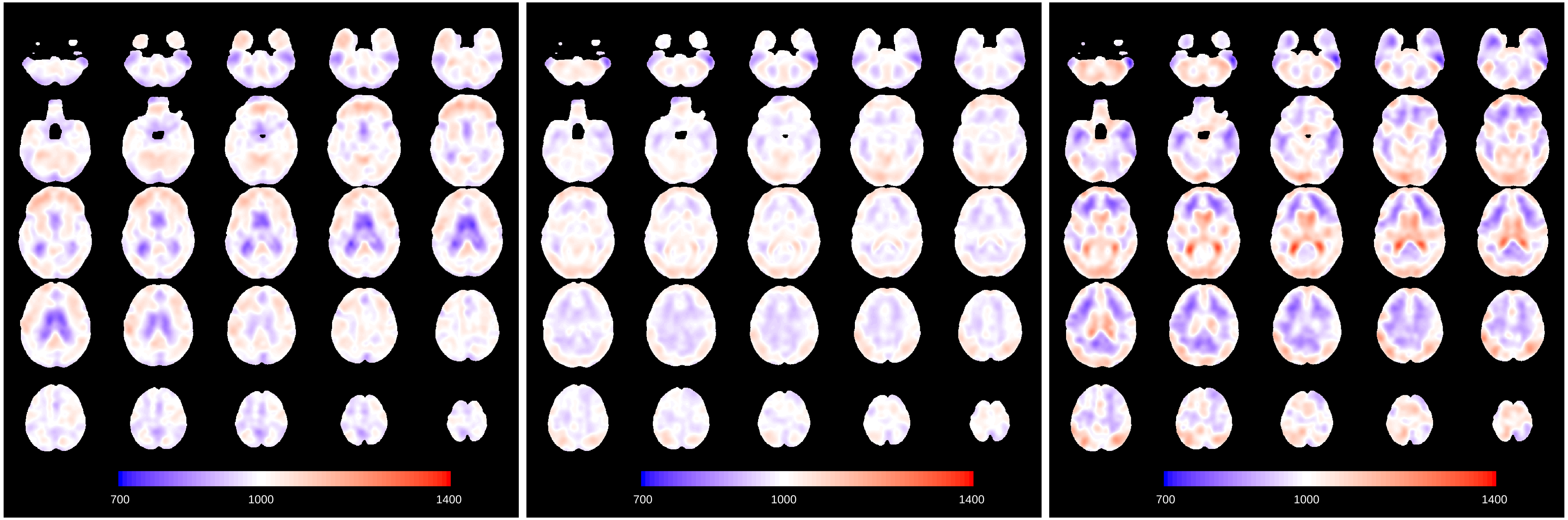

Figure 1: Axial slices of the mean for females at 70 years (left), 80 years (centre) and 90 years (right) in the normative population. Slices are ordered from bottom to top.
